# Change in body mass index during transition to statutory retirement: an occupational cohort study

**DOI:** 10.1186/s12966-017-0539-2

**Published:** 2017-06-26

**Authors:** Sari Stenholm, Svetlana Solovieva, Eira Viikari-Juntura, Ville Aalto, Mika Kivimäki, Jussi Vahtera

**Affiliations:** 1 Department of Public Health, University of Turku and Turku University Hospital, Turku, Finland; 2 Faculty of Social Sciences (Health Science), University of Tampere, Tampere, Finland; 3 Finnish Institute of Occupational Health, Helsinki, Finland; 4 Department of Epidemiology and Public Health, University College London Medical School, London, UK; 5 Clinicum, Faculty of Medicine, University of Helsinki, Helsinki, Finland

**Keywords:** Body weight, BMI, Weight change, Work exposure, Retirement, Aging, Cohort study

## Abstract

**Background:**

Retirement is a major life transition affecting health behaviors. The aim of this study was to examine within-individual changes in body mass index (BMI) during transition from full-time work to statutory retirement by sex and physical work characteristics.

**Methods:**

A multiwave cohort study repeated every 4 years and data linkage to records from retirement registers. Participants were 5426 Finnish public-sector employees who retired on a statutory basis in 2000–2011 and who reported their body weight one to three times prior to (w_−3_, w_−2_, w_−1_), and one to three times after (w_+1_, w_+2_, w_+3_) retirement.

**Results:**

During the 4-year retirement transition (w_+1_, vs. w_−1_) men showed decline in BMI, which was most marked among men with sedentary work (−0.18 kg/m^2^, 95% CI −.30 to −0.05). In contrast, BMI increased during retirement transition in women and was most marked among women with diverse (0.14 kg/m^2^, 95% CI 0.08 to 0.20) or physically heavy work (0.31 kg/m^2^, 95% CI 0.16 to 0.45). Physical activity during leisure time or commuting to work, alcohol consumption or smoking did not explain the observed changes during retirement transition.

**Conclusions:**

In this study statutory retirement was associated with small changes in BMI. Weight loss was most visible in men retiring from sedentary jobs and weight gain in women retiring from diverse and physically heavy jobs.

**Electronic supplementary material:**

The online version of this article (doi:10.1186/s12966-017-0539-2) contains supplementary material, which is available to authorized users.

## Background

Transition to retirement is an important turning point in life accompanied by substantial increase in time availability and changes in daily routines [1]. Previous research has shown that leisure-time physical activity [2, 3] and total alcohol consumption [4, 5] tend to increase during retirement transition. Since changes in weight are related to changes in energy expenditure (physical activity) and energy intake (food and alcohol intake) or their combination, retirement may also contribute to weight change among older adults. Examining changes in weight and factors predisposing such changes in this life stage is important since both weight gain and weight loss may affect health at older ages. It has been shown, for example, that weight gain can increase risk for morbidity and disability [6–9] while unintentional weight loss may increase the risk for mortality [10]. Intentional weight loss accompanied by increased physical activity, on the other hand, is associated with improved physical functioning in older adults [11].

To date, relatively little is known about the effects of retirement on weight, although it is likely that the effects are heterogeneous and in part depend on the job a person is retiring. It is possible, for example, that retirement from physically strenuous work leads to reduction in total physical activity and thus induces weight gain. On the other hand, persons who retire from sedentary jobs may be able to increase energy consumption by engaging more physical activity throughout the day. In agreement with this, some previous studies suggest that weight gain is more common in people retiring from physically strenuous job or blue-collar occupations [12–18]. However, the results for sedentary work and weight change are conflicting, some studies reporting no weight change and other studies weight loss during retirement transition [12, 14]. Whether the changes in body weight differ in men and women is not properly examined. Moreover, most previous research is based on a single dataset, the Health and Retirement Study from the US. The US represents a country in which pension schemes vary and extended labor-marker participation is often due to financial reasons which differs from many European countries [19]. It is therefore important to examine these questions also in other populations and settings. Accordingly, the aim of the current study is to examine changes in body mass index during years preceding retirement, during retirement transition and after retirement with particular focus on potential differences in weight development by sex and physical work characteristics.

## Methods

### Study population

The data were from the Finnish Public Sector study, an ongoing prospective occupational cohort study with identifiable questionnaire surveys. The eligible population of the original cohort included all employees who had been working for a minimum of 6 months in the participating organizations, which included ten towns and six hospital districts, between 1991 and 2005 (*n* = 151,901) [20]. Nested survey cohorts included all those who were employed by the participating organizations at the time of surveys or had left the organizations after participating in an earlier survey. The first survey of the total personnel in all participating organizations was conducted in 2000–2002 and thereafter repeated at 4-year intervals. The first survey for leavers was conducted in 2005 and thereafter repeated at 4-year intervals. For this study we used data from surveys performed for employees in 2000–2002, 2004 and 2008 and leavers in 2005, 2009 and 2013. Survey data for cohort members were successfully linked to records of the Finnish Centre for Pension’s register (retirement date and type), employers’ records (birth date, sex, occupational title) and comprehensive national health registers (diseases and medication) through unique personal identification codes, which are assigned to all citizens in Finland. The FPS study was approved by the Ethics Committee of the Hospital District of Helsinki and Uusimaa.

Of all the FPS cohort members, we first identified those who were employed and responded to at least one survey in 2000–2002, 2004 or 2008 (*n* = 81,587). Of these employees, 19,058 were awarded their first pension by December 31, 2011, and of these, 9787 persons had responded to at least one survey before and after retirement. We focused on those persons who had retired at the statutory retirement age (i.e. old age retirement) as their first awarded pension scheme (*n* = 5898). Participants who retired part-time (*n* = 1619) or on a health grounds (*n* = 2185) or because of unemployment (*n* = 85) were excluded from this study, because these types of retirement are endogenous and potentially related to the causes that may affect weight (e.g. disease) and thus subject to reverse causation bias.

We centered the data around the retirement date. There were three study waves before retirement (w_−3_, w_−2_, w_−1_), and three waves after retirement (w_+1_, w_+2_, w_+3_). Each successive wave was on average 4 years apart from each other. To be included in this study, the participants had to report their body weight and height in at least two surveys, one immediately before and after transition to statutory retirement (i.e., w_−1_ and w_+1_) (*n* = 5458). Then we excluded underweight persons at pre-retirement (*n* = 22) and those with missing occupational titles (*n* = 10) resulting in an analytic sample of 5426 persons. Thus, depending on the retirement date, participants’ observations came from one of the following alternative set of waves: 1) w_−3_, w_−2_, w_−1_, w_+1_, 2) w_−2_, w_−1_, w_+1_, w_+2_, or 3) w_−1_, w_+1_, w_+2_, w_+3_. The relation of the survey years to the study waves around retirement is demonstrated in supplemental material (Additional file 1: Table S1). On average, these participants provided information on body weight at 3.6 (range 2–4) of the possible four study waves during a follow-up of 8–12 years.

### Assessment of retirement

Data on type and date of retirement were obtained from the Finnish Centre for Pensions, which coordinates all earnings-related pensions for permanent residents in Finland [21]. All gainful employment is insured in a pension plan and accrues a pension; thus the pension data with successful linkage were available for all participants. The start dates for any pension were obtained for all participants from 2000 through 2011, irrespective of the participants’ employment status or workplace at follow-up. According to the public sector Employees’ Pension Act, the *statutory retirement* age was generally from 63 to 65 years until 2005 and 63 to 67 years from 2005 onwards, although some individuals had kept their earlier retirement age from the previous pension act in which pension ages in some occupations were below 63 years (e.g., 60 years for primary school teachers, 58 for practical nurses).

### Assessment of body weight and body mass index

Body weight and height were inquired at each study wave and was recorded in kilograms and centimeters, respectively. Based on this information body mass index (BMI) was calculated at each study wave and BMI was categorized into normal weight (18.5 kg/m^2^ ≤ BMI < 25.0 kg/m^2^), overweight (25–29.9 kg/m^2^ ≤ BMI < 30 kg/m^2^) and obesity class I (30 kg/m^2^ ≤ BMI < 35 kg/m^2^) and obesity class II I (35 kg/m^2^ ≤ BMI) [22].

### Assessment of physical work characteristics

Occupational titles were obtained from the employers’ registers and coded according to the Standard Classification of Occupations 2001 by Statistics Finland [23]. Information on the last occupation preceding retirement (w_−1_) was used and if this was not available, then information from w_−2_ was used. We linked each occupational code to validated gender-specific job exposure matrix (JEM) [24], which was developed by using exposure information from a large nationally representative survey (Health 2000 Study [25]). The matrix includes exposure information for more than 401 occupations or occupational groups coded according to the Classification of Occupations 2001 by Statistics Finland. The classification is based down to the 4-digit level, corresponding to the EU’s classification of occupations (ISCO-88(COM)). In addition, national circumstances have been taken into account by adding 5-digit occupational groups, when necessary. In this study population 244 different occupational titles were present which all could be linked to JEM.

For the current study, two physical work characteristics were used, namely “physical heaviness of work” and “sitting at work”. The dichotomized exposures were based on the following two questions at the Health 2000 Survey: “Does your current job involve heavy physical work, in which you have to lift or carry heavy items, to dig, shovel or pound?” (yes/no) and “Do you in your current work need to sit (work machine or car driving not included) on an average at least five hours a day?” (yes/no). By using information from both items, a composite work indicator was created and labelled as “Sedentary” (sitting but no heavy work), “Diverse” (no sitting and no heavy work” and “Physically heavy” (no sitting but heavy work). There was no one in jobs with both sitting and heavy work.

In addition, socioeconomic position (SES) was defined based on ISCO and categorized into high (ISCO categories 1–2 / e.g. teachers, physicians), intermediate (ISCO categories 3–4 / e.g. registered nurses, technicians) and low (ISCO categories 5–9 / e.g. cleaners, maintenance workers) [23].

### Assessment of covariates

To measure non-occupational physical activity, respondents were asked to estimate their average weekly hours of leisure-time physical activity (including commuting) within the previous year in walking, brisk walking, jogging, and running, or their equivalent activities [26]. The time spent on activity at each intensity level in hours per week was multiplied by the average energy expenditure of each activity, expressed in metabolic equivalent (MET) [2, 27]. Alcohol use was based on self-reported habitual frequency and amount of beer, wine and spirits consumption, which was converted into grams of alcohol per week [28]. Due to its distributional skewness, a logarithmic transformation is used in the analyses. Smoking status was categorized into never, former and current smokers. For the analyses, physical activity, alcohol use and smoking were modelled as a time-variant variables since they may influence on weight change.

Disease status was constructed by taking into account chronic diseases in all pre-retirement waves available. Information on chronic illnesses was obtained on nationwide registers: asthma, diabetes, rheumatoid arthritis and coronary heart disease based on the Social Insurance Institution of Finland’s (SII) Drug Reimbursement Register; depression based on the Finnish Prescription Register kept by SII (ATC code N06A) and cancer based on the Finnish Cancer Registry. In addition information on osteoarthritis was obtained from the questionnaires. For the analyses, participants were categorized as having no disease, one disease and two or more diseases before retirement.

An additional covariate possibly related to body weight is a job strain, which was ascertained using questions from the Job Content Questionnaire (JCQ) [29]. The FPS study surveys included job control and job demands scales from the shorter version of the JCQ [30]. The presence of job strain at pre-retirement (w_−1_) was defined as having high demands and a low control score based on the median values from the year 2000 survey.

### Statistical analyses

Characteristics of the study population before retirement (w_−1_) for men and women are presented as mean values for continuous variables and as proportions for categorical variables. Changes in BMI were assessed using linear regression analyses with generalized estimation equations (GEE). The GEE models control for the intra-individual correlation between repeated measurements using an exchangeable correlation structure and is not sensitive to measurements missing completely at random [31, 32].

We started by examining whether BMI change was different among men and women and since the interaction ‘sex x time’ was statistically significant (*p* < 0.0001), all analyses were conducted separately for men and women. In further analyses the interest was in differences by physical work characteristics and thus the models included ‘work characteristics x time’ interaction term. Time variable in our models indicated time point in respect to retirement.

To examine changes in BMI around the transition to retirement, we constructed three consecutive periods: the pre-retirement period (from w _− 3_ to w _− 2_), the retirement transition (from w _− 1_ to w _+ 1_) and the post-retirement period (from w _+ 2_ to w _+ 3_). The periods were non-overlapping to allow testing whether changes in BMI differed between the pre-retirement period, the retirement transition, and the post-retirement period. The statistical significance of these changes were tested using ‘Period x Time’ interaction term where Time was treated as continuous variable.

Adjusted mean estimates and their 95% confidence intervals were calculated to represent an average of 4-year change of BMI at different periods. In addition, risk of obesity (BMI ≥ 30 kg/m^2^) was examined with log-binomial GEE models by calculating risk ratios (RR) within each period by comparing prevalence of obesity at the latter study wave to the previous study wave (e.g. wave _+1_ vs. wave _−1_). The analyses were adjusted for retirement age, SES, time-varying physical activity, alcohol consumption and smoking as well as marital status, number of chronic diseases, job strain and BMI before retirement (w_−1_).

Finally, we conducted a sensitivity analyses among those whom data on BMI were available from all four measurements (*n* = 3683). This was done to address the question on whether including those participants who had missing data in one or two measurement points affected the results. The SAS 9.4 Statistical Package was used for all of the analyses (SAS Institute Inc., Cary, NC).

## Results

The average retirement age differed between sexes being 62.2 (SD 2.3) years in men and 61.8 (SD 1.9) years in women. At the survey before retirement (w_−1_), prevalence of normal weight was 35% in men and 46% in women, overweight 47% in men and 39% in women and obesity 18% in men and 15% in women. Of the participants 13% of men and 16% of women were engaged in heavy physical work and 37% of men and 14% of women had sedentary work. The detailed characteristics in men and women before retirement (w_−1_) are shown in Table 1.

**Table 1:** Characteristics of the study population before retirement in men and women (*n* = 5426)

	Men		Women		
	*n* = 1116		*n* = 4310		*p*-value
	Mean	SD	Mean	SD	
Retirement age (years)	62.2	2.3	61.8	1.9	<.0001
	n	%	n	%	
Retirement age					
< 60	159	14	505	12	<.0001
60–64	758	68	3253	75	
> 64	202	18	559	13	
Socioeconomic status					<.0001
High	537	48	1537	36	
Intermediate	212	19	1229	29	
Low	367	33	1528	36	
Work characteristics					<.0001
Sedentary	414	37	623	14	
Diverse	555	50	2984	69	
Physically heavy	147	13	703	16	
Body mass index					<.0001
Normal weight	384	35	1980	46	
Overweight	522	47	1673	39	
Obese class I	162	15	520	12	
Obese class II	41	4	125	3	
Low leisure-time physical activity^a^					
No	647	58	2491	58	0.99
Yes	463	42	1787	42	
Alcohol use^b^					<.0001
None	85	8	767	18	
Moderate	908	82	3223	75	
Heavy	113	10	298	7	
Smoking status					<.0001
Never	586	55	3361	79	
Former	337	32	546	13	
Current	149	14	322	8	
Presence of chronic diseases					<.0001
0	46	4	224	5	
1	671	60	2220	51	
> 1	399	36	1866	43	
Job strain					<.0001
No	945	85	3086	73	
Yes	164	15	1159	27	

^a^ Low physical activity at leisure time or during commuting to work is defined as less than 14 MET-hours per week. ^b^ Moderate alcohol use corresponds <192 g / week in women and <288 g / week in men and heavy alcohol use ≥192 g / week in women and ≥288 g / week in men.

**Fig. 1:**
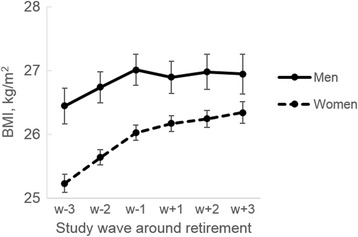
Changes in body mass index during retirement transition in men and women. Adjusted for retirement age and socioeconomic status

Development of BMI before, during and after retirement in men and women are illustrated in Fig. 1. The magnitude of change in BMI within each period differed between pre-retirement, retirement transition and post-retirement periods both in men and women (period x time interaction *p* = 0.0009 for men and *p* < 0.0001 for women). In men BMI increased during pre-retirement period by 0.29 kg/m^2^ (95% confidence interval (CI) 0.13 to 0.46), decreased during retirement transition by 0.11 kg/m^2^ (95% CI -0.22 to −0.01) and became steady during post-retirement period (−0.03 kg/m^2^, 95% CI -0.23 to 0.17) (Table 2). In women BMI increased during pre-retirement period by 0.41 kg/m^2^ (95% CI 0.31 to 0.51) and during the retirement transition by 0.15 kg/m^2^ (95% CI 0.10 to 0.20), but became steady during post-retirement period (0.10 kg/m^2^, 95% CI -0.02 to 0.22) (Table 2). Further adjustments for time-varying physical activity, alcohol consumption and smoking as well as marital status, number of chronic diseases, body mass index and job strain before retirement (w_−1_) did not alter the results.

**Table 2:** Change in body mass index before, during and after retirement transition by physical work characteristics in men

		Time in relation to retirement								
		Pre-retirement (w_−2_ vs. w_−3_)			Retirement transition (w_+1_ vs. w_−1_)			Post-retirement (w_+3_ vs. w_+2_)		
		Mean change^a^	95% CI		Mean change^a^	95% CI		Mean change^a^	95% CI	
Total	Model 1	0.29	0.13	0.46	−0.11	−0.22	−0.01	−0.03	−0.23	0.17
(*n* = 1116)	Model 2	0.30	0.12	0.44	−0.11	−0.22	−0.01	0.07	−0.14	0.28
Sedentary	Model 1	0.21	−0.04	0.46	−0.18	−0.30	−0.05	−0.0004	−0.29	0.29
(*n* = 414)	Model 2	0.26	0.003	0.52	−0.19	−0.32	−0.07	0.08	−0.23	0.38
Diverse	Model 1	0.44	0.20	0.68	−0.05	−0.20	0.11	0.01	−0.28	0.31
(*n* = 555)	Model 2	0.40	0.14	0.65	−0.03	−0.18	0.12	0.13	−0.16	0.43
Physically heavy	Model 1	0.07	−0.41	0.55	−0.20	−0.63	0.23	−0.34	−1.01	0.32
(*n* = 147)	Model 2	0.06	−0.51	0.63	−0.19	−0.64	0.26	−0.22	−0.91	0.46

Data are centered at retirement: w_−1_, w_−2_, and w_−3_ refer to survey waves before retirement, and w_+1_, w_+2_ and w_+3_ refer to survey waves after retirement. ^a^ Change is estimated over 4 years of time. Model1: adjusted for retirement age and socioeconomic position Model 2: Model 1 + adjusted for physical activity, alcohol use and smoking as time-varying covariates and marital status, body mass index, number of chronic diseases and job strain before retirement.

**Fig. 2:**
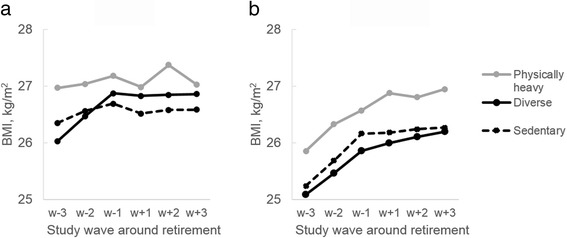
Changes in body mass index during retirement transition in men and women by physical work characteristics. Adjusted for retirement age and socioeconomic status. **a**) Men, **b**) Women

Changes in BMI by work characteristics, were also examined. Men with physically heavy work had slightly higher BMI (27.18 kg/m^2^, 95% CI 26.64 to 27.72) compared to men with sedentary (26.69 kg/m^2^, 95% CI 26.31 to 27.08) and diverse work (26.87 kg/m^2^, 95% CI 26.55 to 27.20) before retirement (w_−1_) (Table 2, Fig. 2a). After adjusting for covariates, men in sedentary work increased their BMI during pre-retiment period (0.26 kg/m^2^, 95% CI 0.003 to 0.52) and decreased during retirement transition (−0.19 kg/m^2^, 95% CI -0.32 to −0.07), but no change was observed during post-retirement period. Among men with diverse and physically heavy work, no significant changes were observed during retirement transition and post-retirement periods.

The results for women are shown in Table 3 and Fig. 2b. Before retirement (w_−1_), women with physically heavy work had higher BMI (26.57 kg/m^2^, 95% CI 26.28 to 26.86) than women with sedentary work (26.16 kg/m^2^, 95% CI 25.82 to 25.50) and with diverse work (25.85 kg/m^2^, 95% CI 25.71 to 26.00). BMI increased in all work characteristics groups during pre-retirement period by about 0.40 kg/m^2^. However, only women with diverse and physically heavy work, showed increase in BMI during retirement transition period (0.16 kg/m^2^, 95% CI 0.09 to 0.22 and 0.30 kg/m^2^, 95% CI 0.15 to 0.46). No statistically significant change in BMI in post-retirement period was observed in any of the work characteristics groups.

**Table 3:** Change in body mass index before, during and after retirement transition by physical work characteristics in women

		Time in relation to retirement								
		Pre-retirement (w_−2_ vs. w_−3_)			Retirement transition (w_+1_ vs. w_−1_)			Post-retirement (w_+3_ vs. w_+2_)		
		Mean change^a^	95% CI		Mean change^a^	95% CI		Mean change^a^	95% CI	
Total	Model 1	0.41	0.31	0.51	0.15	0.10	0.20	0.10	−0.02	0.22
(*n* = 4310)	Model 2	0.41	0.30	0.52	0.16	0.11	0.22	0.11	−0.01	0.23
Sedentary	Model 1	0.45	0.23	0.67	0.02	−0.12	0.16	0.03	−0.37	0.43
(*n* = 623)	Model 2	0.47	0.24	0.71	0.02	−0.12	0.16	0.03	−0.38	0.43
Diverse	Model 1	0.38	0.26	0.50	0.14	0.08	0.20	0.10	−0.04	0.23
(*n* = 2984)	Model 2	0.40	0.27	0.52	0.16	0.09	0.22	0.09	−0.05	0.23
Physically heavy	Model 1	0.47	0.16	0.78	0.31	0.16	0.45	0.14	−0.12	0.41
(*n* = 703)	Model 2	0.38	0.04	0.71	0.30	0.15	0.46	0.25	−0.04	0.53

Data are centered at retirement: w_−1_, w_−2_, and w_−3_ refer to survey waves before retirement, and w_+1_, w_+2_ and w_+3_ refer to survey waves after retirement. ^a^ Change is estimated over 4 years of time. Model 1: adjusted for retirement age and socioeconomic position. Model 2: Model 1 + adjusted for physical activity, alcohol use and smoking as time-varying covariates and marital status, body mass index, number of chronic diseases and job strain before retirement

In addition to changes in absolute BMI values, the risk of obesity within each period was examined. In men, retirement did not increase risk of becoming obese, but women in diverse (RR 1.15, 95% CI 1.07 to 1.22) and physically heavy work had increased risk of obesity during retirement transition (RR 1.20, 95% CI 1.07 to 1.34) (Additional file 1: Table S2).

Finally, we repeated the analyses including only participants who had all four body weight measurements available. The results were very similar to those of the actual analyses with only minor differences in the estimates.. In general, these findings suggest no major selection by sex or physical work characteristics (Additional file 1: Table S3).

## Discussion

The results of this longitudinal study of Finnish public sector workers suggest that transition to statutory retirement is associated with a small decrease in BMI in men and a slight increase in women. In men the decline during retirement transition was most marked among those with sedentary jobs. In contrast, in women the weight increase was most pronounced among those with diverse and physically heavy work. They were also more likely become obese during retirement transition.

These findings add to existing evidence on retirement and weight. An advantage of the present investigation over previous studies is that we examined changes in weight in men and women taking into account both physical work characteristics and SES. In addition, we controlled for a number of underlying factors for weight change, including leisure-time physical activity, alcohol consumption and smoking. Since most of the previous studies have analyzed men and women together [13, 14], our comparison to other studies is limited to studies showing results separately for men or women.

Our findings are consistent with the Health and Retirement Study (HRS) [15] from the US in which retirement was associated with weight gain among women from blue collar occupations, whereas no change was observed in men. The magnitude of average weight change was relatively similar among women in the Finnish Public Sector study (0.8 kg over 4 years) and that HRS study (about 0.5 kg over 2 years). Our findings are also in agreement with an analysis based on the US HRS study by Goldman et al. [12] suggesting that men retiring from sedentary jobs lost about 0.12 BMI-units (kg/m^2^) over 2 years compared to 0.2 BMI-units over 4 years in the present study. Our findings are in contrast to those reported by Goldman et al. [12], Godard et al. [17] and Nooyens et al. [16] as in these studies weight gain among men retiring from physically heavy work was observed. In fact, in our study there was, if anything, a tendency towards decreasing BMI among those with physically heavy work. Taken together, the changes in BMI we observed during retirement transition are statistically significant but in absolute terms relatively small, less than ±1 kg per 4 years. A 5% weight loss or weight gain (3.5 kg for a person who weight 70 kg) is often considered clinical relevant [33]; with this criterion retirement appears not to affect weight status in a clinically relevant way. At the population level, however, even relatively small changes in the distribution of risk factors may relate to considerable changes in benefits and harms in absolute terms as the majority of incident cases of disease occur in people with a medium risk [34, 35].

We also sought to examine the role of underlying factors for weight change by including time-variant leisure-time physical activity, alcohol consumption and smoking in our models. However, none of these factors explained the observed changes during retirement transition. A further important aspect in energy balance is nutrition and eating habits, which were not assessed in this study. In a previous smaller study from the Netherlands, eating habits partially explained changes in men’s weight during retirement transition [16]. Further research is needed to examine whether retirement is associated with changes in nutrition and eating habits and whether this could explain the observed changes in body weight.

Strengths of our study include the repeated measurement of body weight around an objectively determined retirement transition which enabled us to estimate body weight development before, during and after retirement. All participants retired from full-time work to full-time retirement, thus removal of work-related exposures and increase in leisure-time was similar for all. There are some limitations to our study. This study relied on self-reported body weight which is subject to recall and information bias, possibly resulting in under-reporting of body weight [36, 37]. However, there is no reason to assume that these biases would be different for the different retirement periods. The generalizability of the findings may be limited as the cohort consisted of relatively healthy public sector employees of European origin in a Scandinavian welfare state with a relatively genereous retirement scheme. In addition, since the focus of our study was on statutory retirement, the results cannot be generalized to other retirement types, such as early retirement due to poor health. Data from the US shows that obese individuals are more likely to seek early retirement benefits than non-obese [38] and it is possible that for them changes in body weight during retirement transition may differ from those entering to statutory retirement.

## Conclusions

This study suggests that statutory retirement is associated with slight weight loss in men retiring from sedentary jobs and a slight weight gain in women retiring from diverse and physically heavy jobs. The magnitude of change in BMI suggests that retirement does not have clinically relevant influence on weight development among older adults.

## Additional file

Additional file 1:
**Table S1.** Study design. Survey years, their relation to the study waves around retirement and the construction of the pre-retirement, retirement transition and post-retirement periods. **Table S2.** Risk of obesity during pre-retirement, retirement transition and post-retirement periods in men and women. **Table S3.** Change in body mass index before, during and after retirement transition in men and women. Only participants with four observations are included (*n* = 3949). (DOCX 20 kb)
