# Supplementary material for: Change in body mass index during transition to statutory retirement: an occupational cohort study
Source: Int J Behav Nutr Phys Act. 2017 Jun 26;14:85. doi: 10.1186/s12966-017-0539-2 (PMC5485586; doi:10.1186/s12966-017-0539-2)
Supplement: Additional file 1: — Table S1. Study design. Survey years, their relation to the study waves around retirement and the construction of the pre-retirement, retirement transition and post-retirement periods. Table S2. Risk of obesity during pre-retirement, retirement transition and post-retirement periods in men and women. Table S3. Change in body mass index before, during and after retirement transition in men and women. Only participants with four observations are included (n = 3949). (DOCX 20 kb) [file 12966_2017_539_MOESM1_ESM.docx]

**ADDITIONAL FILE 1**

**Table S1. Study design. Survey years, their relation to the study waves around retirement and the construction of the pre-retirement, retirement transition and post-retirement periods.**

**Table S2. Risk of obesity during pre-retirement, retirement transition and post-retirement periods in men and women.**

**Table S3. Change in body mass index before, during and after retirement transition in men and women. Only participants with four observations are included (n=3,949).**

**Table S1. Study design. Survey years, their relation to the study waves around retirement and the construction of the pre-retirement, retirement transition and post-retirement periods.**

| **Pre-retirement period** | | **Retirement transition** | | | **Post-retirement period** | |
| --- | --- | --- | --- | --- | --- | --- |
| **wave_-3_** | **wave_-2_** | **wave_-1_** | **RETIREMENT** | **wave_+1_** | **wave_+2_** | **wave_+3_** |
| *n*=1,336 | *n*=3,290 | *n*=5,426 |  | *n*=5,426 | *n*=2,971 | *n*=1,145 |
|  |  | 2000-2002 |  | 2005 | 2009 | 2013 |
|  | 2000-2002 | 2004 |  | 2009 | 2013 |  |
| 2000-2002 | 2004 | 2008 |  | 2013 |  |  |

**Table S2. Risk of obesity during pre-retirement, retirement transition and post-retirement periods in men and women.**

|  | Time in relation to retirement | | | | | | | | |
| --- | --- | --- | --- | --- | --- | --- | --- | --- | --- |
|  | Pre-retirement (w_-2_ vs. w_-3_) | | | Retirement transition (w_+1_ vs. w_-1_) | | | Post-retirement (w_+3_ vs. w_+2_) | | |
|  | RR* | 95% CI | | RR * | 95% CI | | RR * | 95% CI | |
| **Men** |  |  |  |  |  |  |  |  |  |
| Total (n=1116) | 1.14 | 0.93 | 1.40 | 0.93 | 0.84 | 1.03 | 1.05 | 0.88 | 1.25 |
| Sedentary (n=414) | 1.11 | 0.86 | 1.42 | 0.88 | 0.75 | 1.03 | 1.11 | 0.83 | 1.48 |
| Diverse (n=555) | 1.27 | 0.83 | 1.93 | 0.96 | 0.84 | 1.10 | 1.01 | 0.77 | 1.31 |
| Physically heavy (n=147) | 0.94 | 0.70 | 1.25 | 0.98 | 0.71 | 1.35 | 1.06 | 0.73 | 1.55 |
|  |  |  |  |  |  |  |  |  |  |
| **Women** |  |  |  |  |  |  |  |  |  |
| Total (n=4310) | 1.15 | 1.01 | 1.31 | 1.15 | 1.09 | 1.21 | 1.00 | 0.92 | 1.09 |
| Sedentary (n=623) | 1.18 | 0.89 | 1.57 | 1.10 | 0.96 | 1.26 | 0.76 | 0.55 | 1.04 |
| Diverse (n=2984) | 1.17 | 0.99 | 1.38 | 1.15 | 1.07 | 1.22 | 1.02 | 0.92 | 1.13 |
| Physically heavy (n=703) | 1.09 | 0.78 | 1.52 | 1.20 | 1.07 | 1.34 | 1.08 | 0.90 | 1.29 |

Notes: Data are centered at retirement: w_-1_, w_-2_, and w_-3_ refer to survey waves before retirement, and w_+1_, w_+2_ and w_+3_ refer to survey waves after retirement. * Risk ratio (RR) is calculated within each period by comparing prevalence of obesity at the latter study wave to the previous study wave. Models are adjusted for retirement age, socioeconomic status, physical activity, alcohol use and smoking as time-varying covariates and marital status, number of chronic diseases and job strain before retirement.

**Table S3. Change in body mass index before, during and after retirement transition in men and women. Only participants with four observations are included (n=3,683).**

|  | Time in relation to retirement | | | | | | | | |
| --- | --- | --- | --- | --- | --- | --- | --- | --- | --- |
|  | Pre-retirement (w_-2_ vs. w_-3_) | | | Retirement transition (w_+1_ vs. w_-1_) | | | Post-retirement (w_+3_ vs. w_+2_) | | |
|  | Mean change* | 95% CI | | Mean change* | 95% CI | | Mean change* | 95% CI | |
| **Men** |  |  |  |  |  |  |  |  |  |
| Total (n=722) | 0.30 | 0.12 | 0.47 | -0.08 | -0.22 | 0.06 | 0.08 | -0.13 | 0.29 |
| Sedentary (n=274) | 0.27 | 0.02 | 0.53 | -0.18 | -0.34 | -0.03 | 0.09 | -0.21 | 0.40 |
| Diverse (n=367) | 0.38 | 0.12 | 0.64 | 0.05 | -0.13 | 0.24 | 0.14 | -0.16 | 0.43 |
| Physically heavy (n=81) | 0.11 | -0.46 | 0.68 | -0.32 | -1.10 | 0.45 | -0.23 | -0.95 | 0.49 |
|  |  |  |  |  |  |  |  |  |  |
| **Women** |  |  |  |  |  |  |  |  |  |
| Total (n=2961) | 0.41 | 0.31 | 0.52 | 0.18 | 0.11 | 0.25 | 0.11 | -0.01 | 0.23 |
| Sedentary (n=424) | 0.49 | 0.26 | 0.72 | 0.03 | -0.13 | 0.19 | 0.06 | -0.33 | 0.45 |
| Diverse (n=2072) | 0.39 | 0.27 | 0.51 | 0.19 | 0.11 | 0.27 | 0.09 | -0.05 | 0.23 |
| Physically heavy (n=465) | 0.41 | 0.08 | 0.75 | 0.28 | 0.09 | 0.48 | 0.24 | -0.04 | 0.53 |

Notes: Data are centered at retirement: w_-1_, w_-2_, and w_-3_ refer to survey waves before retirement, and w_+1_, w_+2_ and w_+3_ refer to survey waves after retirement. * Change is estimated over four years of time. Models are adjusted for retirement age, socioeconomic status, physical activity, alcohol use and smoking as time-varying covariates and marital status, body mass index, number of chronic diseases and job strain before retirement.
